# Clinical Outcomes of Total En Bloc Spondylectomy for Previously Irradiated Spinal Metastases: A Retrospective Propensity Score-Matched Comparative Study

**DOI:** 10.3390/jcm12144603

**Published:** 2023-07-11

**Authors:** Noriaki Yokogawa, Satoshi Kato, Takaki Shimizu, Yuki Kurokawa, Motoya Kobayashi, Yohei Yamada, Satoshi Nagatani, Masafumi Kawai, Takaaki Uto, Hideki Murakami, Norio Kawahara, Satoru Demura

**Affiliations:** 1 Department of Orthopaedic Surgery, Graduate School of Medical Sciences, Kanazawa University, Kanazawa 920-8641, Japan; 2 Department of Orthopaedic Surgery, Nagoya City University Graduate School of Medical Sciences, Nagoya 467-8601, Japan; 3 Department of Orthopaedic Surgery, Kanazawa Medical University, Kahoku 920-0293, Japan

**Keywords:** total en bloc spondylectomy, spinal metastasis, radiotherapy history, propensity score matching, postoperative complication, local recurrence, overall survival

## Abstract

This study aimed to investigate the clinical outcomes of total en bloc spondylectomy (TES) for spinal metastases previously treated with radiotherapy (RT). This study enrolled 142 patients who were divided into two groups: those with and those without an RT history. Forty-two patients were selected from each group through propensity score matching, and postoperative complications, local recurrence, and overall survival rates were compared. The incidence of postoperative complications was significantly higher in the group with an RT history than in the group without an RT history (57.1% vs. 35.7%, respectively). The group with an RT history had a higher local recurrence rate than the group without an RT history (1-year rate: 17.5% vs. 0%; 2-year rate: 20.8% vs. 2.9%; 5-year rate: 24.4% vs. 6.9%). The overall postoperative survival tended to be lower in the group with an RT history; however, there was no significant difference between the two groups (2-year survival: 64.3% vs. 66.7%; 5-year survival: 47.3% vs. 57.1%). When planning a TES for irradiated spinal metastases, the risk of postoperative complications and local recurrence should be fully considered.

## 1. Introduction

The spine is the most common site of bone metastasis [1], with 20–40% of patients with cancer having spinal metastases, up to 20% of which become symptomatic [2,3,4]. Intolerable pain and paralysis due to spinal metastases can reduce the performance status (PS) and quality of life of patients with cancer, with a potential impact on life expectancy. While advances in systemic therapies, such as chemotherapy, molecularly targeted biologics, and immunotherapy, have dramatically improved the life expectancy of such patients, they have limited efficacy against bone metastases [5]. Therefore, as with the treatment for the primary tumor, long-term local control of spinal metastases and the prevention of skeletal-related events are of great importance.

In the 1990s, Tomita et al. developed total en bloc spondylectomy (TES) for complete surgical resection of spinal tumors with a reduced risk of local tumor recurrence and complications [6,7]. TES is expected to not only provide long-term local control of spinal metastases but also improve the prognosis of some carcinomas [8,9,10]. In particular, bone metastases from kidney and thyroid cancers and low-grade sarcomas are highly resistant to radiation, and surgical resection of isolated metastatic lesions has been accepted in previous studies or guidelines [11,12,13]. Therefore, solitary spinal metastases from these malignancies are good indications for TES, and favorable outcomes have been reported even in patients with concomitant pulmonary metastases [14,15,16].

Meanwhile, radiotherapy (RT) is widely used as the standard treatment for spinal metastases, and its effectiveness is indisputable. However, the duration of its efficacy is often limited [17], and tumors may recur after RT in long-term survivors, requiring spinal surgery owing to severe pain and paralysis. Recent advances in systemic treatments have prolonged the life expectancy of patients with cancer; thus, the use of TES for managing tumor recurrence after irradiation is expected to increase.

Perioperative complications are generally more likely to occur during surgery at the irradiated sites and have also been shown to increase in TES for irradiated spinal metastases [18]. Furthermore, TES at the irradiated sites reportedly increases local recurrence in the peridural region [19]. Thus, there is a concern that TES for irradiated spinal metastases may not only increase postoperative complications, but also increase local recurrence with a potential effect on survival. However, to the best of our knowledge, no previous studies have examined the effects of an RT history on these series of clinical outcomes in TES. This study aimed to compare the postoperative complications, local recurrence, and overall survival rates after TES in patients with spinal metastases with and without an RT history, after background adjustment with propensity score matching.

## 2. Materials and Methods

### 2.1. Study Design and Setting

This was a single-center retrospective cohort study. Prior to the study, the institutional review board of our university approved the study protocol for examining the patients’ charts and radiographic images without the need for individual informed consent (approval number: 2015-075).

### 2.2. Study Participants

We retrospectively reviewed patients with spinal metastases who underwent TES at our university hospital between 2005 and 2018. TES was indicated based on the following criteria: solitary and removable spinal lesions (tumors involving three or fewer contiguous vertebrae), an Eastern Cooperative Oncology Group (ECOG) PS score of three or less, and a stable disease with no or limited other metastases. No postoperative adjuvant RT for TES was administered. Follow-up continued every 6–12 months until the patient died. An outpatient computed tomography (CT) scan of the chest to the pelvis was performed routinely. Patients underwent magnetic resonance imaging to confirm the presence of a recurrent tumor if the CT results indicated local tumor recurrence in the surgical lesion. Patients who could not be followed up with within 2 years after the surgery were excluded from the study. 

### 2.3. Variables and Outcomes

The variables included patient demographics (the age at the time of surgery, sex, a history of treatment for spinal metastases [RT and surgery], level of spinal metastases, presence of an epidural extension, severity of spinal cord paralysis [Frankel grade], ECOG PS score, presence of other bone metastases or major organ metastases, and revised Tokuhashi score [20]) and surgical characteristics (the number of vertebrae removed, operation time, and intraoperative blood loss). Postoperative complications requiring medical intervention, postoperative local recurrence, and postoperative survival were investigated as clinical outcomes.

### 2.4. Statistical Analysis

Patients were divided into two groups based on a history of RT. Quantitative data are presented as the mean ± standard deviation. Intergroup differences for continuous variables were examined using a student’s *t*-test for parametric data and the Mann–Whitney U test for nonparametric data, whereas categorical data were compared using the chi-squared test.

A propensity score-matching analysis was performed to compare the postoperative complications, local recurrence, and overall survival rates in patients with and without a history of RT. Propensity score-matching is widely used in retrospective cohort studies to control confounding bias [21]. In this study, a propensity score was calculated for each variable. No statistical sample size calculations were conducted.

Postoperative local recurrence and overall survival rates were determined using Kaplan–Meier curves and compared between the groups using the log-rank test. In addition, for all patients, independent factors associated with overall survival were identified using the Cox proportional hazards model with a forced entry method. The appropriate number of independent variables was estimated based on the number of events, and in addition to the age at the time of surgery, sex, and radiation history, the following key parameters that have been previously reported to influence the outcomes of surgery for spinal tumors were included as independent variables: the history of local surgery, ECOG PS score, revised Tokuhashi score, level of spinal metastases, and the number of resected vertebrae [14,16,22,23,24,25,26]. The results are shown as hazard ratios and 95% confidence intervals. The statistical significance was set at *p* < 0.05. Statistical analyses were performed using the SPSS software (version 23; IBM Corp., Armonk, NY, USA).

## 3. Results

### 3.1. Patients and Surgical Characteristics

In total, 152 patients underwent TES for spinal metastasectomy at our hospital from 2005 to 2018, of whom 142 were followed up with for >2 years after the surgery (follow-up rate: 93.4%). Of these, 46 (32.4%) had a history of RT and 96 (67.6%) had no such history. The patients with an RT history had undergone TES for recurrence or progression of spinal metastases following RT, with no planned RT as a neo-adjuvant therapy for TES. The average irradiation dose for RT was 41.5 Gy, and the average time from irradiation to surgery was 21.0 months. Among the patients’ backgrounds and surgical factors, a larger number of those in the group with an RT history had a significantly poorer PS score, revised Tokuhashi scores, and a higher number of resected vertebrae (Table 1).

Forty-two pairs of patients were selected with propensity score matching. There were no significant differences among the groups for each variable (Table 2).

### 3.2. Postoperative Complication Rate

The incidence of postoperative complications was significantly higher in the group with an RT history than in the group without an RT history (57.1% vs. 35.7%), and the mean number of complications per case was significantly higher in the group with an RT history (1.3 vs. 0.6). Individual complications such as wound dehiscence, surgical site infection, cerebrospinal fluid leakage, and respiratory complications were more likely to occur in the group with an RT history, but there were no significant differences between the groups (Table 3). No patients had surgery-related deaths in both groups.

### 3.3. Postoperative Local Recurrence Rate

**Figure 1:**
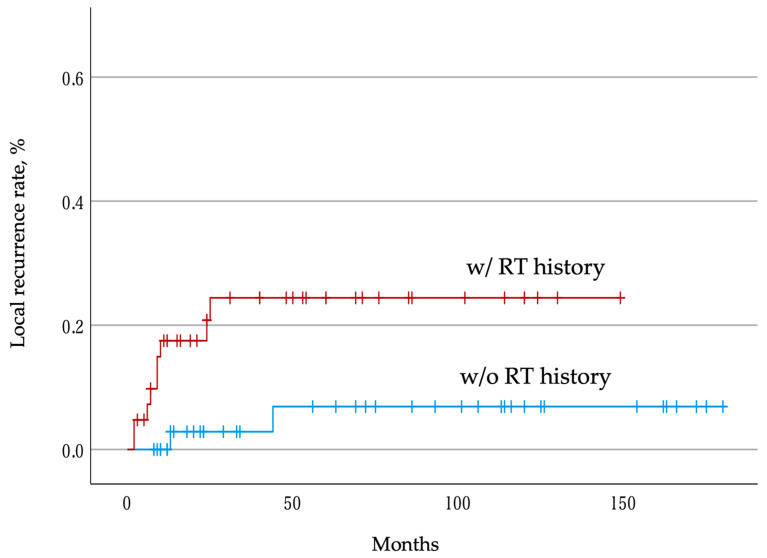
Postoperative local tumor recurrence rate. Kaplan–Meier curves showing the local tumor recurrence rate in patients with and without an RT history. The tick marks indicate the last date of follow-up. RT, radiotherapy.

Postoperative local recurrences were observed in nine of 42 patients (21.4%) in the group with an RT history and in two of 42 patients (4.8%) in the group without an RT history at the time of the last follow-up. In the Kaplan–Meier analysis, the 1-year, 2-year, and 5-year local recurrence rates in the group with an RT history were 17.5%, 20.8%, and 24.4%, respectively, whereas the 1-year, 2-year, and 5-year local recurrence rates in the group without an RT history were 0%, 2.9%, and 6.9%, respectively. Although the estimated median local recurrence time was not reached in both groups, the log-rank test revealed that patients with an RT history had a higher local recurrence rate than those without such a history (*p* = 0.019) (Figure 1).

### 3.4. Overall Postoperative Survival Rate

**Figure 2:**
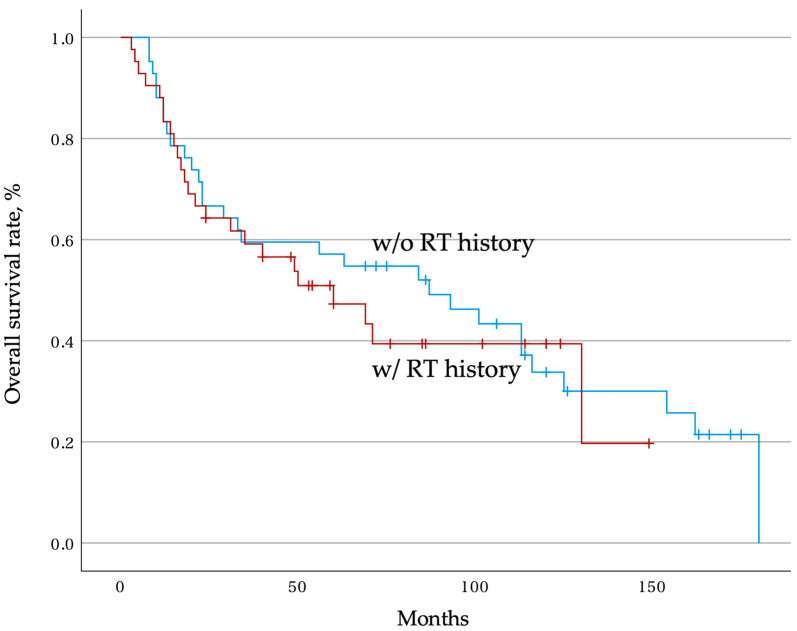
Overall postoperative survival rate. Kaplan–Meier curves show the overall postoperative survival rate of patients with and without an RT history. The tick marks indicate the last date of follow-up. RT, radiotherapy.

The overall postoperative survival rates were 64.3% at 2 years and 47.3% at 5 years in the group with an RT history, showing a slightly lower trend than 66.7% and 57.1% at 2 and 5 years, respectively, in the group without an RT history, but the difference was not significant (*p* = 0.62). The estimated median survival time was 60 months (95% confidence interval: 25.0–95.0) in the group with an RT history and 87 months (95% confidence interval: 36.7–137.3) in the group without an RT history (Figure 2).

### 3.5. Prognostic Factor

The Cox proportional hazards model revealed that only the revised Tokuhashi score was a significant prognostic factor and that a history of RT and prognosis were not significantly associated (Table 4).

## 4. Discussion

Modern oncology offers a multitude of treatment options for patients with spinal metastases, including effective systemic therapy, radiation therapy, and surgery. SBRT (stereotactic body radiation therapy) has recently become the mainstay treatment for long-term control of spinal metastases in solitary or isolated metastatic disease [5,27]. Studies with large cohorts have reported 1-year local control rates of 72–90% for spinal metastases from various types of primary cancers [28,29,30,31,32]. However, TES in large, experienced centers reportedly shows lower local recurrence rates than SBRT [33,34]. Furthermore, a recent study comparing TES and separation surgery with postoperative stereotactic radiosurgery reported that while both were efficient treatments for patients with isolated spinal metastases and spinal cord compression, local tumor control was better in the TES group [35]. Therefore, TES is considered to have the highest local curative effect on spinal metastases. 

With recent advances in cancer treatment, patients with irradiated spinal metastases with potentially long-term prognoses increasingly require further treatments [36]. In this TES case series, one-third of the patients had recurrent or advanced spinal metastases following RT, and such a situation is likely to continue. TES remains technically demanding because of the anatomical spine features surrounding the spinal cord and nerve roots and their proximity to major blood vessels, and prior irradiation can make the procedure even more difficult. In addition, these patients are often at an advanced stage of disease and may have had multiple prior systemic therapies, which may put them at a high risk for undergoing major spinal surgery [36]. However, evidence regarding the efficacy and safety of TES for irradiated spinal metastases is lacking; therefore, it is important to determine the clinical outcomes of TES in previously irradiated patients. The results of this study, such as postoperative complications and local recurrence rates after TES for irradiated spinal metastases, would provide significant information for patient counseling and for determination of the indication for TES.

Radiation injuries generally occur in normal tissues at the irradiated site, resulting in a high incidence of wound complications. In the case of decompression and fusion surgery for spinal metastases in patients with a history of RT, the incidence of wound complications, such as wound dehiscence, reportedly exceeds 30%, which is more than three times that of non-irradiated cases [18,37]. Radiation-induced microvascular damage and decreased collagen production owing to fibroblast dysfunction are thought to be involved in wound dehiscence [38]. Furthermore, the incidence of a dural injury and cerebrospinal fluid (CSF) leakage associated with TES after RT is reportedly very high [18,39,40]. In basic research conducted in our department, fibrosis of the epidural space and thinning of arachnoid barrier cells have been observed as chronic effects in irradiated mouse spines [41]. Fibrosis in the epidural space causes adhesions between the dura and surrounding tissue, which may contribute to dural injuries during dissection. In addition, the thinning of the arachnoid barrier cells responsible for meningeal permeability may spur CSF leakage. Similar changes are thought to occur around the pleura adjacent to the spine, particularly in TES at the thoracic spine level, where pleural injuries during the detachment of adhesions and associated pleural effusions are more likely to occur.

In this study, the complication rates after TES were compared between patients with and without an RT history in a matched background, and the results were consistent with previous findings that complications were more common in patients with an RT history. In contrast, comparisons of individual complications, such as delayed wound healing, wound infection, and spinal fluid leakage, showed no significant differences between the groups, although they were more common in those with an RT history. There are two possible reasons for this. One is the insufficient sample size. The other reason is that we routinely administered prostaglandin E_1_ postoperatively in accordance with a previous report [42], which may have led to fewer complications. However, the complication rate of TES at the irradiated site remains high, and further efforts to reduce complications and fully explain the risk of complications in patients are needed.

This study also showed a significantly increased local recurrence rate in TES for irradiated spinal metastases. Radiation-induced malignant transformation of tumor cells has recently been a focus of interest. Tumor cells that become resistant to irradiation are known to exhibit increased cellular physiological activities such as invasion, migration, and adhesion [43,44,45], which may possibly lead to postoperative local recurrence. Normally, tumor invasion into the dura mater is rare because the dura mater acts as a barrier against tumor extension [46]. However, in our basic study with a mouse model of tumor spinal cord compression, we confirmed that the collagen fibers composing the dura mater in irradiated mice were disorganized following degeneration, and defects were observed on the surface of the dura mater, leading to a significant increase in tumor invasion into the dura mater [47]. These disruptions in the dural barrier mechanisms may lead to intradural invasion of post-RT spinal tumors and contribute to local recurrences after TES. In addition, as mentioned above, radiation can cause adhesions in the epidural space [41], which may make complete resection of the spinal tumor more difficult. Therefore, when conducting TES for spinal metastases in patients with an RT history, the risk of postoperative local recurrence should be carefully considered, and furthermore, the surgical plan should be based on a careful review of imaging for intradural tumor invasion. Most importantly, TES should be preferred over RT as an initial treatment whenever possible, especially for spinal metastases from renal and thyroid cancers or low-grade sarcomas for which metastatic resection is recommended, resulting in fewer postoperative complications and long-term local control.

In our case series, no significant difference in overall survival after TES was found between patients with and without prior RT, although there was a trend toward a worse prognosis in the group with an RT history. Undeniably, this was influenced by the insufficient sample size. However, since only the revised Tokuhashi score was identified as a prognostic factor in this study, multiple factors, such as PS score, cancer type, and major organ metastases, may play a role in postoperative overall survival. In addition, although long-term local control with total en bloc spondylectomy may be helpful in prolonging life expectancy, the effectiveness of the postoperative systemic treatment presumably contributes significantly to the overall survival [35]. In other words, a history of RT does not preclude a long-term prognosis after TES. Therefore, it is considered acceptable to apply TES in patients with isolated spinal metastases who are expected to have a long-term prognosis, regardless of a history of RT. However, the risk of postoperative complications and local recurrence must be considered in TES at irradiated sites, and an alternative treatment may need to be selected, depending on the situation. Some reports recommend re-irradiation via SBRT for irradiated spinal metastases with 1-year local control rates of 15–25% [27,48]. In contrast, the local control rate 1 year after TES with a prior RT history in this study was 17.5%. Direct comparisons are difficult because the study cohorts are not identical in characteristics, and the long-term outcomes of re-irradiation with SBRT are still unclear; therefore, further studies are needed to make these comparisons.

The limitations of this study include its single-center and retrospective nature with no statistical sample size calculations. Furthermore, there were variations in the dose, method, and timing of RT, as well as in the cancer type, and the effect of postoperative systemic therapy was not considered. The subgroup analyses were not included in this study because of the limited sample size. Therefore, a larger sample size is essential to make our findings more practical.

## 5. Conclusions

In TES for spinal metastases, the postoperative complication and local recurrence rates were significantly higher in patients with an RT history than in those without an RT history. The overall postoperative survival tended to be lower in patients with an RT history, although there was no significant difference compared to those without an RT history.

## Figures and Tables

**Table 1:** Comparison of patient demographics and surgical characteristics with and without an RT history.

	w/ RT History (*n* = 46)	w/o RT History (*n* = 96)	*p*-Value
Age at the time of surgery (years), mean ± SD	56.3 ± 11.2	57.1 ± 10.4	0.7
Sex: male, *n* (%)	26 (56.5)	54 (56.3)	0.98
Primary tumor	renal:17, thyroid:4, breast:4, lung:6, others:13, unknown:2	renal:30, thyroid:13, breast:13, lung:7, others:30, unknown:3	0.72
History of local surgery, *n* (%)	5 (10.9)	4 (4.2)	0.15
Level of spinal metastases: lumbar, *n* (%)	12 (26.1)	30 (31.3)	0.53
Epidural extension, *n* (%)	40 (87.0)	72 (75.0)	0.1
Frankel grade A–C, *n* (%)	15 (32.6)	18 (18.8)	0.07
ECOG PS score ≥ 3, *n* (%)	16 (39.1)	18 (18.8)	0.04 *
Other bone metastases, *n* (%)	21(45.7)	32 (33.3)	0.16
Major organ metastases, *n* (%)	12 (26.1)	33 (34.4)	0.32
Revised Tokuhashi score, mean ± SD	10.3 ± 2.2	11.3 ± 2.2	0.02 *
Number of resected vertebrae, mean ± SD	1.8 ± 0.8	1.4 ± 0.7	0.02 *
Operative time (min), mean ± SD	545.9 ± 182.6	496.0 ± 134.7	0.11
Intraoperative bleeding (mL), mean ± SD	869.2 ± 941.1	826.9 ± 942.1	0.81

RT, radiotherapy; SD, standard deviation; ECOG PS, Eastern Cooperative Oncology Group performance status; * *p* < 0.05.

**Table 2:** Comparison of the adjusted variables between the propensity score-matched groups.

	w/ RT History (*n* = 42)	w/o RT History (*n* = 42)	*p*-Value
Age at the time of surgery (years), mean ± SD	57.0 ± 10.4	55.5 ± 11.1	0.52
Sex: male, *n* (%)	23 (54.8)	25 (59.5)	0.66
Primary tumor	renal:15, thyroid:3, breast:4, lung:6, others:12, unknown:2	renal:18, thyroid:4, breast:4, lung:4, others:11, unknown:1	0.99
History of local surgery, *n* (%)	4 (9.5)	2 (4.8)	0.4
Level of spinal metastases: lumbar, *n* (%)	12 (28.6)	18 (42.9)	0.17
Epidural extension, *n* (%)	36 (85.7)	37 (88.1)	0.75
Frankel grade A–C, *n* (%)	12 (28.6)	9 (21.4)	0.45
ECOG PS score ≥ 3, *n* (%)	13 (31.0)	10 (23.8)	0.46
Other bone metastases, *n* (%)	17(40.5)	15 (35.7)	0.65
Major organ metastases, *n* (%)	11 (26.2)	14 (33.3)	0.47
Revised Tokuhashi score, mean ± SD	10.5 ± 2.0	10.8 ± 2.2	0.65
Number of resected vertebrae, mean ± SD	1.7 ± 0.8	1.6 ± 0.8	0.6
Operative time (min), mean ± SD	546.1 ± 189.0	573.3 ± 118.3	0.44
Intraoperative bleeding (mL), mean ± SD	903.0 ± 975.5	1144.6 ± 965.3	0.26

RT, radiotherapy; SD, standard deviation; ECOG PS, Eastern Cooperative Oncology Group performance status.

**Table 3:** Postoperative complication rates.

	w/ RT History (*n* = 42)	w/o RT History (*n* = 42)	*p*-Value
Postoperative complication, *n* (%)	24 (57.1)	15 (35.7)	0.04 *
Number of complications, mean ± SD	1.3 ± 1.3	0.6 ± 0.8	0.02 *
Wound dehiscence, *n* (%)	7 (16.7%)	3 (7.1%)	0.31
Surgical site infection, *n* (%)	5 (11.9%)	1 (2.4%)	0.2
Cerebrospinal fluid leakage, *n* (%)	8 (19.0%)	2 (4.8%)	0.09
Respiratory, *n* (%)	10 (23.8%)	3 (7.1%)	0.07
Cardiovascular, *n* (%)	4 (9.5%)	2 (4.8%)	0.68
Gastrointestinal, *n* (%)	3 (7.1%)	1 (2.4%)	0.62
Neurological, *n* (%)	9 (21.4%)	6 (14.3%)	0.39

RT, radiotherapy; SD, standard deviation; * *p* < 0.05.

**Table 4:** Hazard ratio of mortality after total en bloc spondylectomy.

Variables	HR	95%CI	*p*-Value
Age at the time of surgery (years)	1.01	0.98–1.03	0.69
Sex: male	0.93	0.59–1.46	0.76
History of radiotherapy	1.18	0.72–1.91	0.51
History of local surgery	0.94	0.37–2.38	0.9
ECOG PS score	1.02	0.77–1.36	0.87
Revised Tokuhashi score	0.79	0.70–0.89	<0.001 *
Level of spinal metastases: lumbar	1.46	0.59–2.47	0.16
Number of resected vertebrae	1.14	0.82–1.58	0.43

HR, Hazard ratio; CI, confidence interval; ECOG PS, Eastern Cooperative Oncology Group performance status; * *p* < 0.05.

## Data Availability

The datasets used and analyzed in the current study are available from the corresponding author upon reasonable request.
